# Analyzing the Impact of Traffic Congestion Mitigation: From an Explainable Neural Network Learning Framework to Marginal Effect Analyses

**DOI:** 10.3390/s19102254

**Published:** 2019-05-15

**Authors:** Jianping Sun, Jifu Guo, Xin Wu, Qian Zhu, Danting Wu, Kai Xian, Xuesong Zhou

**Affiliations:** 1 School of Traffic and Transportation, Beijing Jiaotong University, Beijing 100044, China; sunjp2004@126.com (J.S.); 16120903@bjtu.edu.cn (D.W.); 2 Beijing Transport Institute, Beijing 100073, China; xiank@bjtrc.org.cn (K.X.); xzhou74@asu.edu (X.Z.); 3 School of Sustainable Engineering and the Built Environment, Arizona State University, Tempe, AZ 85281, USA; 4 School of Transportation and Logistics, Southwest Jiaotong University, Chengdu 611756, China; 15928665871@163.com

**Keywords:** computational graph, traffic demand estimation, congestion mitigation, marginal analyses, TensorFlow

## Abstract

Computational graphs (CGs) have been widely utilized in numerical analysis and deep learning to represent directed forward networks of data flows between operations. This paper aims to develop an explainable learning framework that can fully integrate three major steps of decision support: Synthesis of diverse traffic data, multilayered traffic demand estimation, and marginal effect analyses for transport policies. Following the big data-driven transportation computational graph (BTCG) framework, which is an emerging framework for explainable neural networks, we map different external traffic measurements collected from household survey data, mobile phone data, floating car data, and sensor networks to multilayered demand variables in a CG. Furthermore, we extend the CG-based framework by mapping different congestion mitigation strategies to CG layers individually or in combination, allowing the marginal effects and potential migration magnitudes of the strategies to be reliably quantified. Using the TensorFlow architecture, we evaluate our framework on the Sioux Falls network and present a large-scale case study based on a subnetwork of Beijing using a data set from the metropolitan planning organization.

## 1. Introduction

As the population size, economic growth, and personal travel activities continue to increase, traffic congestion in metropolitan areas remains one of the major concerns for urban transportation planning and management agencies [1]. Recurring traffic congestion is often caused by a regional imbalance involving excess demand and limited infrastructure, while nonrecurring congestion is mainly caused by various traffic incidents and severe weather conditions. Many advanced intelligent transportation systems (and more recently, smart transportation initiatives) have been developed to enable more sophisticated traffic demand management and reliable selection of effective congestion mitigation strategies.

The emerging traffic big data environment brings both opportunities and challenges to the core functions of traffic demand estimation and control. For example, Toole et al. (2015) demonstrated progress in using sampled mobile phone data to infer the most traveled routes, which are extremely difficult to discover from traditional survey data [2]. By mining information from different data sources (e.g., traffic count sensors, mobile phone service records, floating car data, and automatic vehicle identification (AVI) data), planners and decision-makers hope to gain deep insight into human mobility patterns, which will accordingly increase the confidence level and reduce the uncertainty of transportation strategy evaluations [3,4,5,6,7,8]. Over the past several decades, many researchers have highlighted the need for a fully integrated connection from big data sources to pattern recognition and the deployment of final traffic demand/control strategies [9,10,11,12].

While it is important to extract and visualize spatial and temporal patterns from diverse data sources collected from advanced sensor networks, transportation planners and managers are more concerned with how to gain insight into the different causes of congestion because the benefits of different urban traffic congestion mitigation strategies must be better quantified in a data-rich environment. This expectation requires transformative advances not only within the traditional domains of traffic behavioral analysis and demand estimation, but also within the emerging field of big data itself [13,14]. Moreover, how to develop an explainable deep learning framework has been identified as one of the top 10 key challenges for the AI community [15].

**Figure 1:**
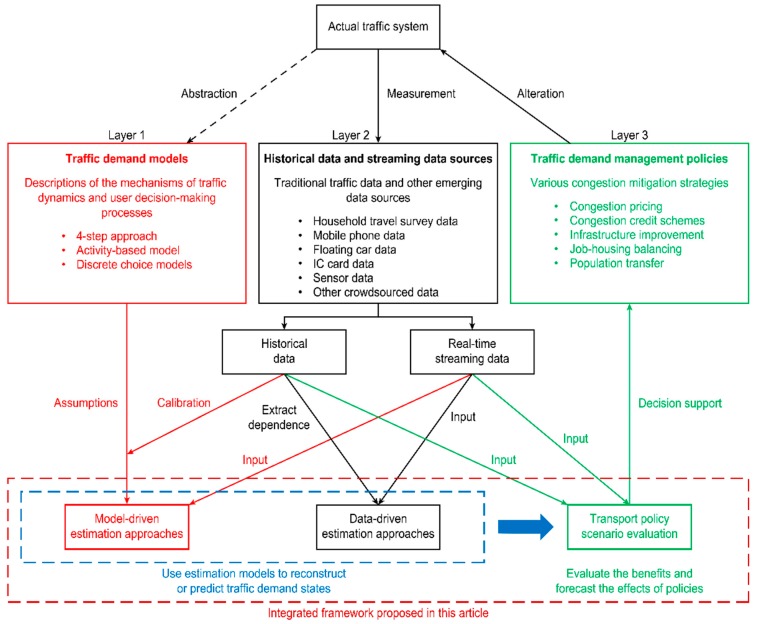
Three basic layers of a traffic demand management system.

Based on the above research needs, we attempt to bridge two important gaps between the three functional layers in a data-centric transportation planning and management framework: Models, data, and policy scenario evaluation, as shown in Figure 1.

1. The gap between layers 1 and 2 is related to the wide variety of available traffic data sources and the fact that core traffic network models are typically difficult to calibrate consistently.
2. The gap between layers 2 and 3 lies in the fact that traffic models contain many elements with limited certainty, whereas mission-critical scenario evaluation requires reliable current-state estimates and policy-sensitive forecasts.

## 2. Literature Review

A traffic demand management system is characterized by three modules: Traffic demand models, different data sources, and traffic demand management policies (e.g., congestion mitigation policies), as displayed in Figure 1. Both model-driven and data-driven approaches provide estimates of traffic demand states for evaluating the effects of congestion mitigation strategies in different scenarios.

### 2.1. Model-Driven Travel Demand Estimation Approaches

The first module includes conventional model-driven approaches for traffic demand estimation that are motivated by a priori knowledge of the kinds of decision-making that must be performed while traveling [16,17]. Traditionally, these decision-making processes are conceived as four-step choices, including trip generation, trip distribution, mode-split models, and traffic assignment [18]. Individual route choice behavior is assumed based on the user equilibrium condition, in which each user chooses the optimal route that minimizes his or her travel costs [19]. Discrete choice models are also used to estimate the modes and route choice behaviors of users [20]. Recently, a number of researchers have attempted to develop activity-based models to estimate users’ trip chains [21] (see Figure 1).

One model-driven approach that focuses on network-wide demand estimation and prediction of origin-destination (OD) demand and route choice probabilities, as well as the resulting traffic network flow patterns, is based on “link proportions” obtained through static and dynamic traffic assignment (STA/DTA). However, in most of the literature, the four-step process and demand estimation are independent. Some studies simply combine STA/DTA with OD matrix estimation (ODME) using bi-level programming models [22,23,24], where the lower level is an STA/DTA model and the upper-level model uses econometric estimators to obtain estimates of the OD matrix.

It should be noted that the real-world deployment of model-driven methods faces technical and computational barriers. Different types of models are not consistent in their formulations of traffic demand. For example, dynamic network assignment for the management of information to travelers (DynaMIT) uses a deterministic queuing and speed model [25], dynamic traffic assignment and simulation for advanced network informatics (DYNASMART) adopts a Greenshield model [26], and the visual interactive system for transportation algorithms (VISTAs) use a cell transmission model [27]. Because the STA/DTA process provides “link proportions” as inputs for demand estimation, different models might lead to different estimates.

Conversely, the accuracy of STA/DTA models also depends heavily on the data available to calibrate the models (see Figure 1). In particular, DTA models require excessive data processing. The input of a DTA model includes demand-side inputs, the OD demand, supply-side inputs, and the network topology. The barriers involve the collection of representative data, the transformation of those data into the DTA input format, and the modeling and evaluation of control strategies.

One task undertaken in this paper is to develop a general framework that integrates the model-driven approach with the traffic demand estimation process using the forward and backward mechanism of computational graphs (CGs). Furthermore, the concept of CGs provides us with a comprehensive learning framework to coordinate the multiple traffic data sources required in traditional four-step methods.

### 2.2. Data-Driven Travel Demand Estimation Approaches

Beyond the use of traditional household survey data in existing models and studies [19,20,28], a number of data-driven traffic demand estimation approaches and online streaming data-driven models have been proposed (see Figure 1) that rely on emerging big data sources [2,28,29,30,31,32,33,34,35].

Inductive loops, radars, and cameras have become the predominant fixed vehicle detection devices in most cities due to their low unit equipment costs and relatively high performance [10,36,37]. Many existing ODME methods focus on the estimation of OD matrices using link counts derived from fixed sensor observations. It has been widely recognized that OD flow patterns are not unique because of the non-uniqueness of the path flows and the limited observation data collected from sensors on links [38,39]. The in-car navigation Global Position System (GPS) technology has matured into a rapidly growing industry. Floating car data have been used in conjunction with link count data and video camera data to derive OD matrices and analyze route choices and trip length distributions [40,41]. Furthermore, mobile phone data make it possible to capture the characteristics of human mobility. Hao et al. [21] applied mobile phone data in activity-based models to generate user tours. Interestingly, Bonnel et al. [42] compared OD matrices that were separately generated from household surveys and mobile phone data and found a large disparity between the two outputs. In the existing literature, secondary data sources from social network services have also been used to study individual travel behaviors [43,44,45].

In data-driven traffic demand estimation models, it is extremely challenging to determine an overall cause–effect explanation based on the partially observable information acquired from multiple data sources. In addition, to achieve the full potential of data-assisted traffic congestion mitigation and demand management, it is critical to have a data mining platform that is sensitive to the requirements of urban planners in a heterogeneous data environment.

From the perspective of big data fusion, Wu et al. [46] proposed the **big data-driven transportation computational graph** (BTCG) framework as a fundamental mathematical modeling tool for performing multilayered traffic demand estimation based on the forward and backward propagation mechanism. The multilayered representation used in this framework structurally models four levels of travel demand variables, including trip generation, OD matrices, path flows, and link flows. Ma et al. (2019) extended the framework to the problem of multiclass traffic demand estimation [47]. This paper further investigates the application of CGs to facilitate data-assisted traffic congestion mitigation and traffic demand management.

### 2.3. Existing Congestion Mitigation Strategies

Traffic congestion can be defined in many ways. Because traffic demands change constantly, city congestion varies from day to day. However, approximately half of all congestion recurs every day in the same location. This paper focuses on investigating such recurrent congestion (see Figure 1). Traffic congestion can be reduced using various mitigation strategies. The most intuitive solution is to build new roadways to add new capacity. However, the famous Braess paradox shows that sometimes, new capacity may decrease utility for users [19]. Moreover, in metropolitan areas, the right-of-way is expensive; consequently, other mitigation options must be considered. The advent of ride-sharing services means that providing high-occupancy vehicle (HOV) lanes for vehicles with two or more passengers is also a feasible solution for expanding capacity.

Congestion pricing strategies [48,49,50] and credit schemes (e.g., tradable credit tickets) [51,52,53,54,55,56] based on market mechanisms are also effective tactics for mitigating congestion. Temporally, variable pricing strategies work to shift peak-hour traffic flows to off-peak periods. Spatially, these strategies incentivize people to consider using public transit or taking alternative routes to their destinations. Various pricing schemes have been investigated in the existing literature, such as trip-rate-based pricing, travel-distance-based pricing, and travel-time-based pricing [56,57,58,59].

Despite the success of congestion pricing in some cities (London has seen benefits from the implementation of congestion pricing in 2003) [60], toll schemes do not appeal to the public. Based on this fact, Wu et al. [61] indicated the importance of calculating the marginal external cost of one extra toll on links because congestion pricing may lead to a possible loss of overall social welfare. Wu et al. [61] recommended a Pareto-improving strategy to increase social benefit without increasing the travel expense for every stakeholder. However, a Pareto-efficient system is not always guaranteed. In most cases, it is important to develop an efficient method of calculating the marginal effect of one extra toll on each link and the tradeoff between benefits and costs.

Traffic congestion is also closely related to urban planning factors, such as job locations, land use, and house (rental) pricing. From the urban planning perspective, to reduce long-term congestion, planners tend to implement population transfers and redesign residential locations and workplaces to balance employment supply and demand [62,63,64,65,66]; however, to the best of our knowledge, very few studies have comprehensively considered the marginal effects of macroscopic policies on the traffic volume on each link.

Another contribution of this paper is to extend the computational graph (CG) based learning framework to evaluate the marginal effects of different congestion mitigation strategies in a big data context.

### 2.4. Outline

The remainder of this paper is organized as follows. Section 3 presents a general description of the overall system architecture and a conceptual illustration based on CGs. In Section 4, we propose a compact description of the traffic demand estimation problem based on the existing CG framework and extend the deep learning framework to the evaluation of the effects of congestion mitigation strategies by mapping those strategies onto CGs. Section 5 reports computational results obtained based on the Sioux Falls network and a subnetwork of Beijing to demonstrate the effectiveness and applicability of our framework. Section 6 concludes the paper and discusses some possible extensions of our work.

## 3. System Architecture and Conceptual Illustration

**Figure 2:**
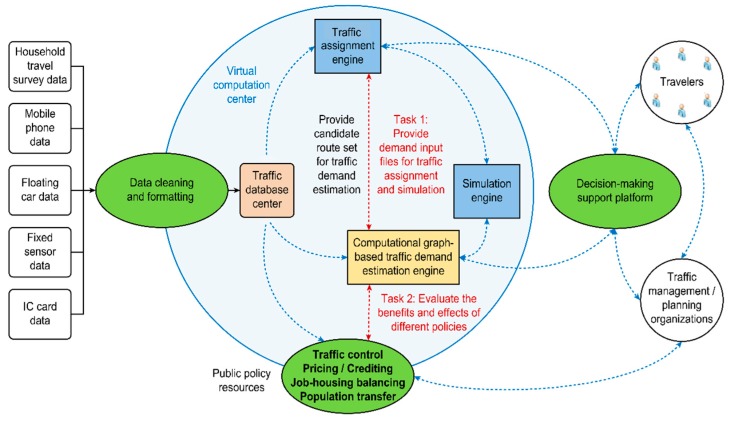
The system architecture of the “super-simulation” system.

As background, in this section, we introduce the overall architecture of the “super-simulation system” that is designed for the new generation of data-driven traffic management platforms. It should be noted that the proposed CG-based learning framework for traffic demand estimation is only one of the engines of this “super-simulation” system. The architecture depicted in Figure 2 reveals the relationship between the CG-based demand estimation engine and the STA/DTA simulation engine.

In addition, we will briefly report on how big data sources and real-world congestion mitigation strategies interact with our traffic demand estimation engine (i.e., the CG-based learning framework). This discussion is followed by a comprehensive review of the concept of a CG and its mathematical relationship to discrete choice models, which shows that the CG representation is sufficiently flexible to be used in formulating various problems in the field of transportation modeling.

### 3.1. System Architecture

The proposed CG-based learning framework is an important component of the overall system, as shown in Figure 2. A central traffic database is established that receives multiple types of traffic data, including household survey data, mobile phone data, floating car data, loop detector data, and data from other possible sources. After being cleaned and formatted, the data are input into the CG-based traffic demand estimation engine and the traffic assignment engine. The outputs of these two engines can be viewed as the input to the simulation engine for visualizing the traffic system [67].

As shown in Figure 2, the CG-based traffic demand estimation engine and the traffic assignment engine (for STA/DTA) provide feedback to each other. On the one hand, the CG-based traffic demand estimation engine provides input files for the traffic assignment engine. On the other hand, the traffic assignment engine initializes the candidate route set for the traffic demand estimation engine. Zhuge et al. [68] reported on how to generate the candidate route set using a tree-based assignment (20 iterations are usually required to arrive at relatively stable conditions; see [68]). The CG-based traffic demand estimation engine should also be fully integrated with the transport policy resources. Then, we will be able to predict the possible demand patterns under different transport policies.

In summary, the essential tasks of the traffic demand estimation engine are as follows:

1. Estimate multilayered traffic demands (i.e., trip generation, trip distribution, and path/link flows).
2. Produce the input file for the traffic assignment engine.
3. Evaluate the effects of various transport policies.

Because this paper focuses on policy analysis, we do not consider the interaction between traffic assignment and traffic demand estimation. We invoke the STA engine only to generate the candidate physical route set. Furthermore, we consider only the private car mode in a regional network during rush hour. The traffic demand estimation engine will be extended in the future.

### 3.2. Computational Graphs and Marginal Effect Analyses

The CG concept serves as a basic description language for many machine learning methods, such as artificial neural networks [69]. In particular, CGs serve as the basis of a fine-grained framework that can be used to decompose complex composite functions into a sequence of nested mappings. Each mapping in a CG (represented by an edge) expresses an elementary operation involving only one or two arguments.

In the field of computer science, CGs provide a paradigm for developing machine learning systems in a big data environment because they enable the efficient and flexible management, operation, and control of data sources using intuitive graphical representations and elementary mathematical operations. Consequently, CGs have become basic building blocks for many current machine learning software packages [70,71,72,73]. For example, TensorFlow, developed by the Google Brain team, is a machine learning library based on a CG framework.

CGs are also related to traditional transportation demand modeling. Below, we present an illustrative example adapted from Koppelman and Bhat (2006) [20] in the context of discrete choice models to show how to formulate a mode-split model (a multinomial logit model) using a CG. We refer readers to a previous introduction to the relationship between CGs and deep learning methods [69]. Furthermore, Zhao et al. (2019) provide a good discussion of the relationship between machine learning and logit modeling [74].

#### 3.2.1. Comparison between Discrete Choice Modeling and Computational Graph Modeling Based on a Mode-Split Model

Consider an example with two available alternatives: Drive alone (DA) and transit (TR). The planners intend to encourage users to use transit services instead of private driving to mitigate congestion in the driving network. The following utility function implies that the decision-maker preferences are a function of the average income (INC) and travel time (TT). For simplicity, we do not consider interaction terms or constant terms:

(1) $$U_{DA}=\beta_{DA,1}INC-\beta_{2}TT_{DA},$$

(2) $$U_{TR}=\beta_{TR,1}INC-\beta_{2}TT_{TR}.$$

Then, the probability of choosing DA can be calculated using the following multinomial logit model:

(3) $$P_{DA}=\frac{exp\left(U_{DA}\right)}{exp\left(U_{TR}\right)+exp\left(U_{DA}\right)}=\frac{1}{1+exp\left(U_{TR}-U_{DA}\right)}.$$

Table 1 shows an example in which $\beta_{DA,1}=0.004$, $\beta_{TR,1}=0$, and $\beta_{2}=0.02$ for an individual from a household with a $50,000 annual income facing travel times of 30 and 50 min for DA and TR, respectively. The utility and probability calculations are shown in Table 1.

As indicated by Goodfellow et al. [65], a logit model for a binary mode choice can be viewed as the simplest type of neural network, with three layers corresponding to two steps of calculations.

1. The first layer is a stack of neurons that express the utility function of the differences between pairs of alternatives for predicting the DA probability:
  (4) $$U_{TR}-U_{DA}=\left(\beta_{TR,1}-\beta_{DA,1}\right)INC-\beta_{2}\left(TT_{TR}-TT_{DA}\right).$$
  Consider $-\beta_{1}=\beta_{TR,1}-\beta_{DA,1}$; then,
  (5) $$U_{TR}-U_{DA}=-\beta_{1}INC-\beta_{2}\left(TT_{TR}-TT_{DA}\right)=-\beta_{1}INC+\beta_{2}TT_{DA}-\beta_{2}TT_{TR}=\beta_{2}x,$$
  where $x=\left(-INC,\;TT_{DA}-TT_{TR}\right)$ can be viewed as a vector that includes both income and travel time.
2. The second layer applies the logistic sigmoid function, $\sigma \left(U_{DA}-U_{TR}\right)$, as an activation function to squeeze the output of the linear utility function into the interval (0, 1).
3. The third layer calculates the probability of choosing DA:
  (6) $$P_{DA}=\sigma \left(U_{DA}-U_{TR}\right)=\frac{1}{1+exp\left(U_{TR}-U_{DA}\right)}=\frac{1}{1+exp\left(-\beta_{1}INC+\beta_{2}TT_{DA}-\beta_{2}TT_{TR}\right)}.$$

**Figure 3:**
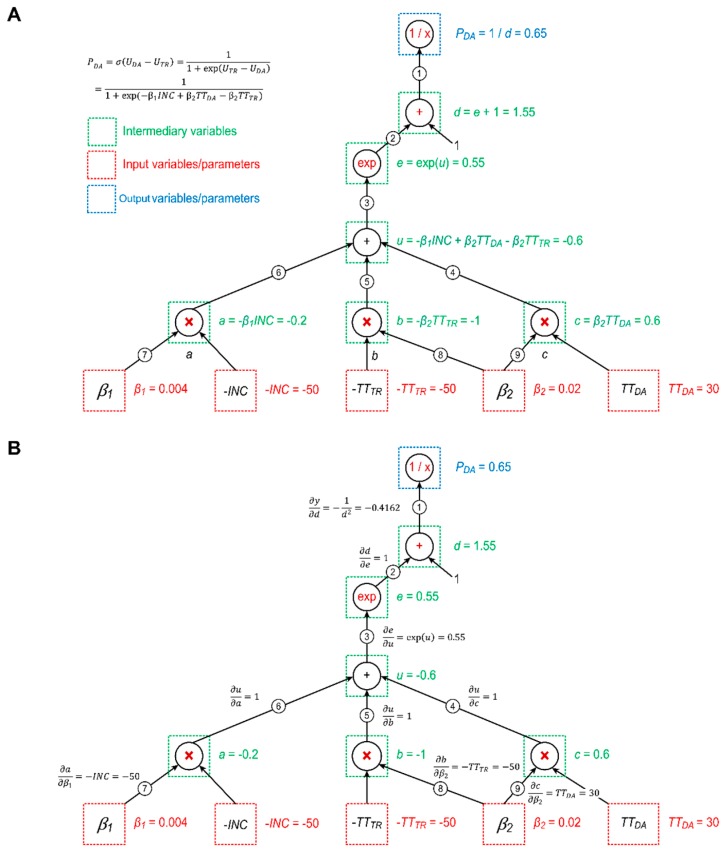
An illustrative computational graph (CG) for a multinomial logit model for the function, $\sigma \left(U_{\mathrm{DA}}-U_{\mathrm{TR}}\right)$; (**A**) Forward propagation of CG; (**B**) Back propagation of CG.

The mode-split model is a composite function consisting of both a linear utility function (Equation (5)) and the sigmoid function (Equation (6)). Figure 3 shows how to describe this function using a CG.

There are seven operations in the expression: Two additions, three multiplications, one exponential operation, and one reciprocal operation. We can describe the mode-split model using the inputs, the outputs, and the following six intermediary variables as vertexes in a CG:

(7) $$a=-\beta_{1}INC,$$

(8) $$b=-\beta_{2}TT_{TR},$$

(9) $$c=\beta_{2}TT_{DR},$$

(10) $$u=a+b+c=-\beta_{1}INC+\beta_{2}TT_{DA}-\beta_{2}TT_{TR},$$

(11) $$e=exp\left(u\right)=exp\left(-\beta_{1}INC+\beta_{2}TT_{DA}-\beta_{2}TT_{TR}\right),$$

(12) $$d=u+1=exp\left(-\beta_{1}INC+\beta_{2}TT_{DA}-\beta_{2}TT_{TR}\right)+1.$$

In the CG plotted in Figure 3, each edge corresponds to a derivative. For example, edge ① corresponds to the partial derivative of $P_{DA}$ with respect to d, and edge ② corresponds to the partial derivative of d with respect to e. We evaluate the output value of $P_{DA}$ by setting the input variables/parameters to certain values and then computing the vertexes progressively through the graph in the upward direction. Let us set $\beta_{1}=0.004$, $\beta_{2}=0.02$, INC=50 thousand dollars, $TT_{DA}=30\;\min$, and $TT_{TR}=50\;\min$. We obtain the same results as in Table 1 using the CG ($P_{DA}=0.65$).

To understand the partial derivatives in these cases, it is important to understand the chain rule in calculus. According to the chain rule, to calculate the partial derivative of $P_{DA}$ with respect to any parameter, we need to sum over all possible paths from vertex $P_{DA}$ to the vertexes of that variable/parameter in the graph while multiplying the derivatives on each edge on the same path. Examples are given below:

1. $\frac{\partial P_{DA}}{\partial \beta_{1}}$ can be calculated by multiplying the partial derivatives on the path ①→②→③→⑥→⑦:
  (13) $$\frac{\partial P_{DA}}{\partial \beta_{1}}=\frac{\partial P_{DA}}{\partial d}\frac{\partial d}{\partial e}\frac{\partial e}{\partial u}\frac{\partial u}{\partial a}\frac{\partial a}{\partial \beta_{1}}=\left(-\frac{1}{d^{2}}\right)exp\left(u\right)\left(-INC\right)=\frac{INCexp\left(-\beta_{1}INC+\beta_{2}TT_{DA}-\beta_{2}TT_{TR}\right)}{{\left[exp\left(-\beta_{1}INC+\beta_{2}TT_{DA}-\beta_{2}TT_{TR}\right)+1\right]}^{2}}.$$
2. $\frac{\partial P_{DA}}{\partial \beta_{2}}$ can be calculated by summing the multiplied partial derivatives on the path ①→②→③→④→⑨ with the multiplied partial derivatives on the path ①→②→③→⑤→⑧:
  (14) $$\begin{array}{c} \frac{\partial P_{DA}}{\partial \beta_{2}}=\frac{\partial P_{DA}}{\partial d}\frac{\partial d}{\partial e}\frac{\partial e}{\partial u}\frac{\partial u}{\partial b}\frac{\partial b}{\partial \beta_{2}}+\frac{\partial P_{DA}}{\partial d}\frac{\partial d}{\partial e}\frac{\partial e}{\partial u}\frac{\partial u}{\partial c}\frac{\partial c}{\partial \beta_{2}}=\left(-\frac{1}{d^{2}}\right)exp\left(u\right)\left(TT_{DA}-TT_{TR}\right)=\\ \frac{\left(TT_{TR}-TT_{DA}\right)exp\left(-\beta_{1}INC+\beta_{2}TT_{DA}-\beta_{2}TT_{TR}\right)}{{\left[exp\left(-\beta_{1}INC+\beta_{2}TT_{DA}-\beta_{2}TT_{TR}\right)+1\right]}^{2}}. \end{array}$$

Typically, “loss errors” can be propagated to update the estimated variables, e.g., $\beta_{1}\;\mathrm{and}\;\beta_{2}$, across the CG using the stochastic gradient descent algorithm. This approach minimizes the sum of the Euclidean residuals for a set of individual observations labeled ${\bar{x}}^{m}=\left({\bar{INC}}^{m},\;{\bar{TT}}_{DA}^{m},\;{\bar{TT}}_{TR}^{m}\right)$ and binary samples, ${\bar{y}}^{m}\in \left\{0,1\right\}$, where m=1,2,…,M:

(15) $$F\left(\beta_{1},\beta_{2}\right)=\underset{\beta_{1},\beta_{2}}{\min}\frac{1}{2}\sum_{m=1}^{M}{\left({\bar{P}}_{DA}^{m}-{\bar{y}}^{m}\right)}^{2}.$$

The gradients, $\frac{\partial F\left(\beta_{1},\beta_{2}\right)}{\partial \beta_{1}}$ and $\frac{\partial F\left(\beta_{1},\beta_{2}\right)}{\partial \beta_{2}}$, can then easily be calculated easily as follows:

(16) $$\frac{\partial F\left(\beta_{1},\beta_{2}\right)}{\partial \beta_{1}}=\sum_{m=1}^{M}\frac{\partial F\left(\beta_{1},\beta_{2}\right)}{\partial {\bar{P}}_{DA}^{m}}\frac{\partial {\bar{P}}_{DA}^{m}}{\partial \beta_{1}},$$

(17) $$\frac{\partial F\left(\beta_{1},\beta_{2}\right)}{\partial \beta_{2}}=\sum_{m=1}^{M}\frac{\partial F\left(\beta_{1},\beta_{2}\right)}{\partial {\bar{P}}_{DA}^{m}}\frac{\partial {\bar{P}}_{DA}^{m}}{\partial \beta_{2}},$$

where $\frac{\partial F\left(\beta_{1},\beta_{2}\right)}{\partial {\bar{P}}_{DA}^{m}}={\bar{P}}_{DA}^{m}-{\bar{y}}^{m}$.

#### 3.2.2. Marginal Effect Analyses Using a Computational Graph

Interestingly, the above formulations can equivalently be used to derive the marginal effect equations obtained with discrete choice models in [19]:

(18) $$\frac{\partial P_{DA}}{\partial \beta_{1}}=\frac{INCexp\left(-\beta_{1}INC+\beta_{2}TT_{DA}-\beta_{2}TT_{TR}\right)}{{\left[exp\left(-\beta_{1}INC+\beta_{2}TT_{DA}-\beta_{2}TT_{TR}\right)+1\right]}^{2}}=INC\;\sigma \left(U_{DA}-U_{TR}\right)\left[1-\sigma \left(U_{DA}-U_{TR}\right)\right],$$

(19) $$\begin{array}{l} \frac{\partial P_{DA}}{\partial \beta_{2}}=\frac{\left(TT_{TR}-TT_{DA}\right)exp\left(-\beta_{1}INC+\beta_{2}TT_{DA}-\beta_{2}TT_{TR}\right)}{{\left[exp\left(-\beta_{1}INC+\beta_{2}TT_{DA}-\beta_{2}TT_{TR}\right)+1\right]}^{2}}\\ \;=\left(TT_{TR}-TT_{DA}\right)\;\sigma \left(U_{DA}-U_{TR}\right)\left[1-\sigma \left(U_{DA}-U_{TR}\right)\right], \end{array}$$

(20) $$\begin{array}{l} \frac{\partial P_{DA}}{\partial TT_{TR}}=\frac{-\beta_{2}exp\left(-\beta_{1}INC+\beta_{2}TT_{DA}-\beta_{2}TT_{TR}\right)}{{\left[exp\left(-\beta_{1}INC+\beta_{2}TT_{DA}-\beta_{2}TT_{TR}\right)+1\right]}^{2}}\\ \;=-\beta_{2}\;\sigma \left(U_{DA}-U_{TR}\right)\left[1-\sigma \left(U_{DA}-U_{TR}\right)\right] \end{array}$$

(21) $$\begin{array}{l} \frac{\partial P_{DA}}{\partial TT_{DA}}=\frac{\beta_{2}exp\left(-\beta_{1}INC+\beta_{2}TT_{DA}-\beta_{2}TT_{TR}\right)}{{\left[exp\left(-\beta_{1}INC+\beta_{2}TT_{DA}-\beta_{2}TT_{TR}\right)+1\right]}^{2}}\\ \;=\beta_{2}\;\sigma \left(U_{DA}-U_{TR}\right)\left[1-\sigma \left(U_{DA}-U_{TR}\right)\right] \end{array}$$

(22) $$\frac{\partial P_{DA}}{\partial INC}=\frac{-\beta_{1}exp\left(-\beta_{1}INC+\beta_{2}TT_{DA}-\beta_{2}TT_{TR}\right)}{{\left[exp\left(-\beta_{1}INC+\beta_{2}TT_{DA}-\beta_{2}TT_{TR}\right)+1\right]}^{2}}=-\beta_{1}\;\sigma \left(U_{DA}-U_{TR}\right)\left[1-\sigma \left(U_{DA}-U_{TR}\right)\right].$$

This relationship implies the potential importance of CGs in economics. Furthermore, the outstanding advantage of CGs is that, when a large number of parameters are involved in a real-world economic system, we can efficiently implement marginal analyses based on the principle of dynamic programming (DP) [69]. The DP principle can be applied in CGs to avoid duplicate calculations and sequentially update the derivatives along the “computing” paths in a backward fashion via the chain rule.

For example, in our case, for all three paths, we can first calculate:

(23) $$\frac{\partial P_{DA}}{\partial d}\frac{\partial d}{\partial e}\frac{\partial e}{\partial u}=\sigma \left(U_{DA}-U_{TR}\right)\left[1-\sigma \left(U_{DA}-U_{TR}\right)\right]=-0.4162\times 1\times 0.55=-0.2284.$$

Then, we can reuse the intermediate result of –0.2284 to calculate the following marginal values:

(24) $$\frac{\partial P_{DA}}{\partial \beta_{1}}=-0.2284\times \left(-50\times 1\right)=11.4217,$$

(25) $$\frac{\partial P_{DA}}{\partial \beta_{2}}=-0.2284\times \left(30-50\right)=4.5687.$$

Provided that we estimate $\beta_{1}=0.004$ and $\beta_{1}=0.02$, we can also use the intermediate result of –0.2284 to calculate several important marginal values:

(26) $$\frac{\partial P_{DA}}{\partial TT_{TR}}=-0.2284\times \left(-0.02\right)=0.0046=0.46\%,$$

(27) $$\frac{\partial P_{DA}}{\partial TT_{DA}}=-0.2284\times 0.02=0.0046=-0.46\%,$$

(28) $$\frac{\partial P_{DA}}{\partial INC}=-0.2284\times \left(-0.004\right)=0.0009=0.09\%.$$

These values imply that if the travel time of transit services decreases by 1 minute, then 0.46% more users will use the transit service system instead of private driving. Thus, the congestion in the driving network can be mitigated. By contrast, if the average income of users increases by 1 dollar, then 0.09% of users will use private driving instead of transit services.

## 4. Congestion Mitigation Strategies Based on the Computational-Graph-Based Learning System

In Section 4.1, Section 4.2 and Section 4.3, we used a compact formulation to rewrite the multilayer CG representation proposed in [46], which captures three steps of the traditional four-step process: Trip generation, trip distribution, and path-flow-based traffic assignment. In contrast to [46], we described the methodology from the perspective of control theories instead of mathematical optimization. In Section 4.4, we attempt to map different congestion mitigation strategies on the CG representation as controllable inputs. Then, the marginal effects of these strategies can be calculated to reflect whether the corresponding control policies can increase the welfare of the transportation system.

### 4.1. Variables

The three basic groups of estimated state variables used in the CG are as follows.

1. Group I: Demand Variables

α indicates the trip generation variable vector, containing the trip generation results from all zones.
q indicates the trip distribution variable vector, containing the flow volume between all OD pairs.
f indicates the path flow variable vector, containing all flow volume on each path in the candidate route set generated by the traffic assignment engine.
v indicates the link flow variable vector, containing the flow volume on each link in the network.

To relate variables, f and v, we define δ as a link-route incidence parameter matrix that indicates whether a route passes through a particular link.

2. Group II: Proportional Variables

γ indicates the OD split rate variable matrix expressing the rate at which each OD pair is selected from each traffic zone.
ρ indicates the route choice proportion variable matrix, expressing the rate at which each route between each OD pair is selected.

3. Group III: Variables in Discrete Choice Models

In the field of traffic modeling, ρ can be determined by a multinomial logit model, which is the simplest type of stochastic network loading algorithm [18]. In the field of deep learning, the multinomial logit model is called the softmax function. The softmax function applies when we wish to represent a probability distribution over a discrete variable with multiple possible values. The softmax function can be regarded as a generalization of the logistic sigmoid function used in the illustrative example in the previous section:

(29) $$\rho_{r}=\frac{exp\left(U_{r}\right)}{\sum_{r\in P\left(w\right)}exp\left(U_{r}\right)}$$

(30) $$U_{p}=-\beta_{w,1}TC_{r}-\beta_{w,2}TT_{r}+\beta_{w}=-\beta_{w,1}\sum_{v\in V\left(p\right)}TC_{v}-\beta_{w,2}\sum_{v\in V\left(p\right)}TT_{v}+\beta_{w},$$

where P(w) represents all candidate routes, r, between OD pairs, and V(r) represents the set of links that are passed through on route r. We express the softmax function (logit model) as follows:

(31) $$\rho =softmax\left(\beta_{1},\beta_{2},\beta ,\;TC,TT\right),$$

where **TC** and **TT** are the travel time parameter vector and the toll parameter vector, respectively.

$U_{r}$ denotes the utility of route r, where
$TC_{r}$ is the toll cost of the route, which is calculated by aggregating the link toll on each related link, and
$TT_{r}$ is the observed travel time of the route, which is calculated by approximately aggregating the observed link travel time for each related link.

In the logit model, we have three estimated variable vectors, as follows:

$\beta_{1}$ indicates the variable vector collecting all $\beta_{w,1}$ between each OD pair, w.
$\beta_{2}$ indicates the variable vector collecting all $\beta_{w,2}$ between each OD pair, w.
β indicates the variable vector collecting all $\beta_{w}$ between each OD pair, w.

### 4.2. System Equations to Express the Computational Graph

**Figure 4:**
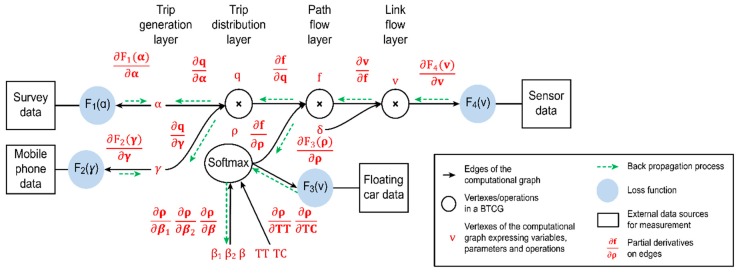
Big data driven transportation computational graph (BTCG) to express a traffic demand estimation model.

The overall traffic demand estimation problem can be represented by the CG $G\left(V_{c},\;A_{c}\right)$ depicted in Figure 4. This CG can be generally viewed as a control system containing the following items.

1. State Variables

$V_{c}$ denotes the set of vertexes, including all state variables of the system, i.e., the estimated variable vectors and matrices listed in Section 4.1, and other parameters that can be expressed as scalars, vectors, matrices, or tensors.

2. State Transitions

$A_{c}$ denotes the set of directed edges that describe different operations in the traffic demand estimation model. If a variable, y, is computed by applying an operation to a variable, x, we draw a directed edge from the vertex representing x to the vertex representing y and annotate the vertex representing y with the name of the operation. Complicated functions are described by combining many elementary operations in a recursive fashion [69].

The graph in Figure 4 expresses the following four constraints to enforce flow conservation and capture user choice behaviors (layer-based state transitions):

(32) α×γ=q,

(33) q×ρ=f,

(34) f×δ=v,

(35) $$\rho =softmax\left(\beta_{1},\beta_{2},\beta ,\;TC,TT\right),$$

where the values of all flow variables are required to be nonnegative. The above constraints can also be viewed as a state transition model in a control system.

### 4.3. Mapping of Data Measurements on the Computational Graph

Traffic demand variable values are propagated forward through the CG in Figure 4 until they reach the output layers, which are connected to external data sources. The outputs of the CG can then be compared to reference measurements to calculate the loss errors. The CG simultaneously uses traffic demand estimation models (e.g., four-step processes, in the field of transportation modeling) and multiple measurements from different data sources to generate estimates of the system’s variables (its states) that are better than the estimates obtainable when using only a single measurement type. As such, CGs can be viewed as a common data fusion method. Furthermore, in the era of big data, we can directly use multiple data samples and the stochastic gradient descent (SGD) algorithm to represent the noise in the system instead of assuming complex probability distributions.

The loss functions used in this paper are as follows:

1. Measurements Associated with Trip Generation, $\bar{\alpha }$

We obtained a reference set of trip generation results for a zone using household survey data. The number of trips associated with this zone can be calculated by multiplying the population size by the rate of trips using private cars [28,75]. The population and trip rate can be calculated using the average numbers for different groups. Then, we have a reference set of trip generation results, $\bar{\alpha }$, and we can obtain the following loss function:

(36) $$F_{1}\left(\alpha \right)=\frac{1}{2M_{1}}∥\alpha -\bar{\alpha }{∥}_{2}^{2},$$

where $M_{1}$ is the number of survey samples.

2. Measurements Associated with the Origin-Destination Split Rates, $\bar{\gamma }$

Reference OD split rates can be generated from raw mobile phone data that match the given zoning system. Because it is technically difficult to identify the mode of transportation using mobile phone data, in this paper, we use mobile phone data to generate the reference OD split rates, $\bar{\gamma }$:

(37) $$F_{2}\left(\gamma \right)=\frac{1}{2M_{2}}∥\gamma -\bar{\gamma }{∥}_{2}^{2},$$

where $M_{2}$ is the number of mobile phone samples. Notably, the locations of cellular base stations are generally not consistent with traffic zones. Hence, some matching assumptions must be made to derive OD splits from cellular records. Furthermore, a rule should also be specified for identifying stationary activities [14,41].

3. Measurements Associated with the Route Choice Proportions, $\bar{\rho }$

Floating car data can be matched to the driving network using matching algorithms [76,77]. Thus, we can obtain reference route choice proportions using floating car data:

(38) $$F_{3}\left(\rho \right)=\frac{1}{2M_{3}}∥\rho -\bar{\rho }{∥}_{2}^{2},$$

where $M_{3}$ is the number of floating car samples. It should be noted that floating car data are sampled data. We should again specify a rule for identifying stationary activities and then match the GPS records to a map using a map matching algorithm (e.g., a hidden Markov chain algorithm) [76,77].

4. Measurements Associated with the Link Flows, $\bar{v}$

Sensor flow counts, $\bar{v}$, are collected from a subset of links:

(39) $$F_{4}\left(v\right)=\frac{1}{2M_{4}}∥v-\bar{v}{∥}_{2}^{2},$$

where $M_{4}$ is the number of sensor data samples. Loop detector data are usually collected at regular time intervals (e.g., every 15 min). In this paper, we sum the counts for all lanes to generate the flow count on each link for each hour.

Overall, the CG can be expressed in terms of the following optimization problem:

(40) $$\min Z=F_{1}\left(\alpha \right)+F_{2}\left(\gamma \right)+F_{3}\left(\rho \right)+F_{4}\left(v\right),$$

s.t. Equations (32)–(35).

Here, we simply assume that all four objective functions are equally important.

### 4.4. Mapping of Congestion Strategies on the Layers of the Computational Graph

**Figure 5:**
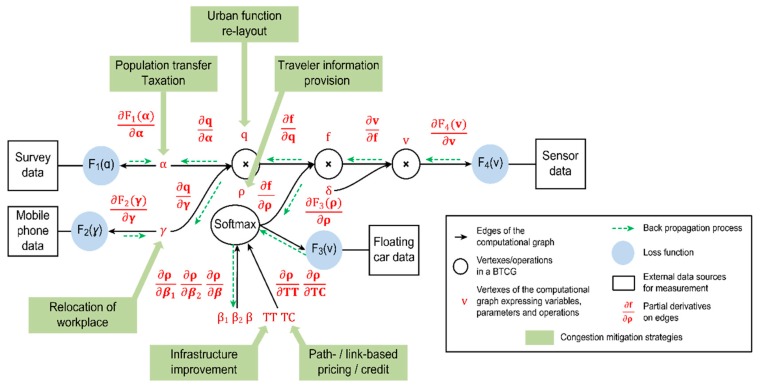
Different congestion mitigation strategies used to control different variables in the CG.

A CG can serve as a helpful tool to support the evaluation of a congestion mitigation strategy. The marginal effects of a policy can be calculated from the partial derivatives of a variable/parameter with respect to all other variables/parameters. That is, the gradients can be viewed as indicators of whether an imposed policy is reasonable. From the perspective of control theories, congestion mitigation strategies can be viewed as controllable inputs acting on traffic demand variables. Figure 5 shows how different strategies map to different variables in the CG. For example, pricing on paths/links can be viewed as controlling the route choice proportions, ρ, or the link flows, **v**. When planners aim to redesign urban layouts or relocate workplaces, the corresponding policies can similarly be viewed as controlling the variables, γ and q. Thus, we can define the **marginal effect** (ME) of such control as follows:

**Definition:**  *The marginal effect (ME) of a congestion mitigation strategy is defined as “the change in the overall negative utility of the system caused by a marginal change in the controlled variable”. In this paper, the negative utility is measured as the total travel time for all users in the transportation network. Let the vector field, F(v), be a link travel time function mapping from ${\mathbb{R}}^{\left|v\right|}$ to ${\mathbb{R}}^{\left|v\right|}$. Then, the ME can be calculated as:*

(41) $$ME=F\left(v+\Delta \right)\cdot {\left(v+\Delta \right)}^{T}-F\left(v\right)\cdot {\left(v\right)}^{T},$$

*where Δ represents the marginal changes in the link flows caused by the control (i.e., the policy). The physical meaning of the ME is the total reduction in “vehicle transport” on all links caused by the policy.*

*1.*  *If ME≤0, the policy decreases the total travel time for users and has a positive effect.*
*2.*  *If ME>0, the policy increases the total travel time for users and has a negative effect.*

One advantage of a CG is that it can directly yield the reciprocal of Δ. Table 2 lists the congestion mitigation strategies considered and the variables they control in the CG. Table 2 also shows how to calculate 1/Δ based on the chain rule in calculus. Each 1/Δ in the table corresponds to a certain “pathway” propagated through the CG, as shown in Figure 4 and Figure 5. The initial values of the variables before learning can be set with rerence to typical historical data.

### 4.5. Algorithm

As shown in Figure 4 and Figure 5, we can obtain a first-order partial derivative on each edge of the CG. Based on these derivatives, the loss errors (gradients) from the measurements are backpropagated to each vertex starting from the outputs. The gradients are calculated using the chain rule. The updating process is widely used in the field of deep learning in the context of the backpropagation (BP) algorithm [69]. In this paper, we implement the algorithm as follows:

**Step** **1.** Estimation:
  **Step** **1.1.** The forward passing step implements trip generation, trip distribution estimation, and traffic assignment.
  **Step** **1.2.** The backward propagation step updates the estimated variables using the SGD algorithm.
  **Step** **1.3.** Steps 1.1. and 1.2. are iteratively implemented until convergence is reached.
**Step** **2.** Evaluation:
  **Step 2.1.** Different mitigation strategies are evaluated by calculating their MEs on link volume.

Because our proposed learning framework can be regarded as a kind of control system, in Table 3, we compare the above algorithm with the standard Kalman filter (KF) (i.e., a linear Gaussian state-space model) [5,78,79,80]. The similarity between them is that they both provide a general framework for state estimation and data fusion. Both algorithms are recursive and follow a two-step process. The prediction step of the KF, corresponding to the forward passing process in Step 1.1 of our CG-based algorithm, produces the current estimates. The update process of the KF, corresponding to the backward propagation in Step 1.2 of our CG-based algorithm, updates the estimates based on the observed measurements. The difference between them is that they use different methods of updating their estimates. While the KF uses the Kalman optimal gain to update the state variables, the above algorithm implements a gradient-based update strategy. Furthermore, the KF applies a linear transition matrix to generate the next-stage estimates and uses covariance matrices to express the correlations between state variables. By contrast, our framework directly uses a CG to describe these relationships. Similar to other deep learning methods, our algorithm uses the SGD to capture the noise in the data, while the KF usually requires an assumed probability distribution.

It is important to note that the above algorithm can be easily implemented using the TensorFlow data programming architecture, which has been widely applied in many machine learning applications. To see the source code of our CG-based learning framework using the TensorFlow Python API, readers can refer to [81].

## 5. Numerical Examples

In this section, we present two numerical examples to illustrate the effectiveness of our methodology.

### 5.1. A Case Study Based on the Sioux Falls Network

We implemented our model based on multiple samples in the Sioux Falls network to validate the effectiveness of our framework. The input data (measurements) included two samples of hypothetical household survey data (trip generation results, $\bar{\alpha }$), two samples of mobile phone data (OD split rates, $\bar{\gamma }$), two samples of floating car data (route choice proportions, $\bar{\rho }$, for some routes), and three samples of sensor data (link flow counts, $\bar{\nu }$). We assumed that the data were collected during one hour of the morning peak time on several working days.

Notably, the accuracy of calibration will depend on the synthesized hypothetical data. To avoid discrepancies between different data sources, we designed one “seed” group of hypothetical data that satisfied the flow conservation constraints of Equations (36)–(39) in an ad hoc manner. The data set included the following:

1. One sample of survey data: Reference trip generation results for 5 zones (i.e., zones 1, 2, 7, 13, and 20).
2. One sample of mobile phone data: Reference OD split rates for 20 OD pairs (i.e., origin zones 1, 2, 7, 13, and 20 and destination zones 9, 11, 22, and 24).
3. One sample of floating car data: We enumerated all candidate paths between the 20 OD pairs, then randomly selected 7 of these paths and adopted assumed route choice proportions for them.
4. One sample of sensor data: We assigned assumed link counts to 7 links.

**Figure 6:**
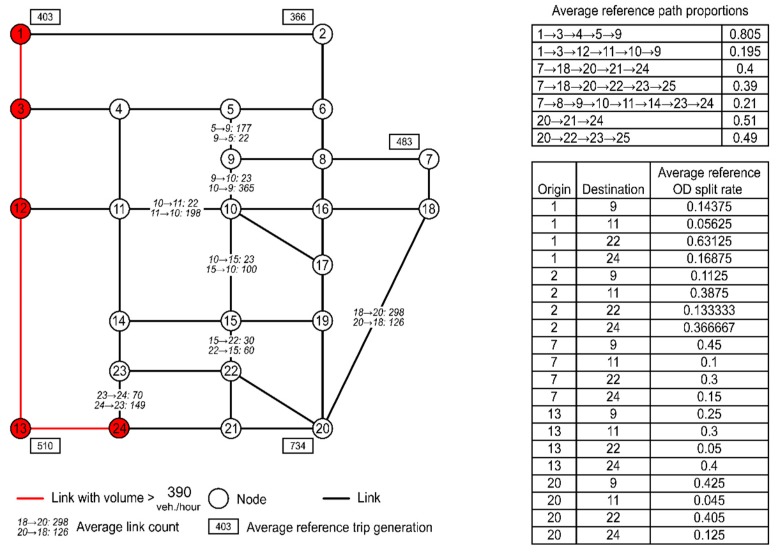
Basic information on the simplified Sioux Falls network (units of link flows: vehicles/hour).

Then, we added some random perturbations to the “seed” samples to generate additional samples. The complete data set can be found in [81]. Figure 6 shows only the average values of our data set. All flows are expressed in units of vehicles/hour.

#### 5.1.1. Calibration Using Multiple Data Sources

**Figure 7:**
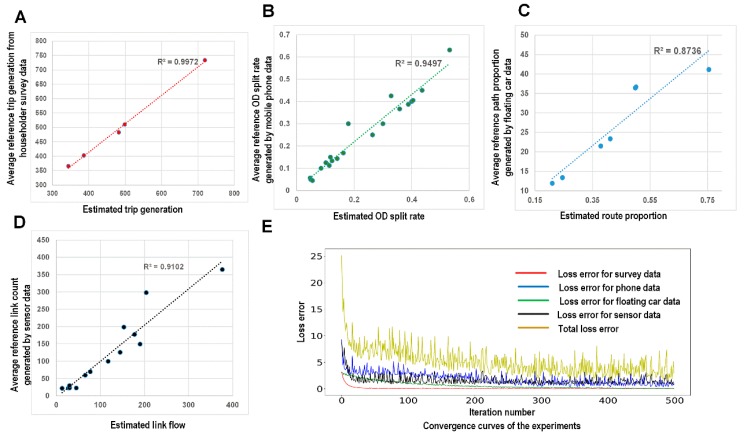
Linear regression lines between the corresponding estimated values and the average reference measurements from the various data sources (**A**) Household data, (**B**) Mobile phone data, (**C**) Floating car data, and (**D**) Sensor data; (**E**) The convergence curves of the loss functions for all data sources (units of link flows: vehicles/hour).

Figure 7 shows the trend lines between the average estimated values and the average reference measurements. We find that reasonably satisfactory R-square values are achieved for all data sources. However, this finding does not imply that our proposed learning framework can always achieve good loss errors. When the inconsistency between different data sets increases, the obtained loss errors will worsen. However, this case study demonstrates that our proposed learning framework can simultaneously decrease the values of all four loss functions between the estimated values and the reference measurements. Figure 7 displays the convergence curves for the four different data sources. Because of the use of the SGD algorithm, the curves fluctuate, reflecting the random noise in the data. Figure 6 also displays four links (links (1, 3), (3, 12), (12, 13), and (13, 24)) with estimated volumes of >390 vehicles/hour (i.e., link flow/link capacity > = 1.3). We find that sensors are not installed on any of these four links.

#### 5.1.2. Analysis of Congestion Components

**Figure 8:**
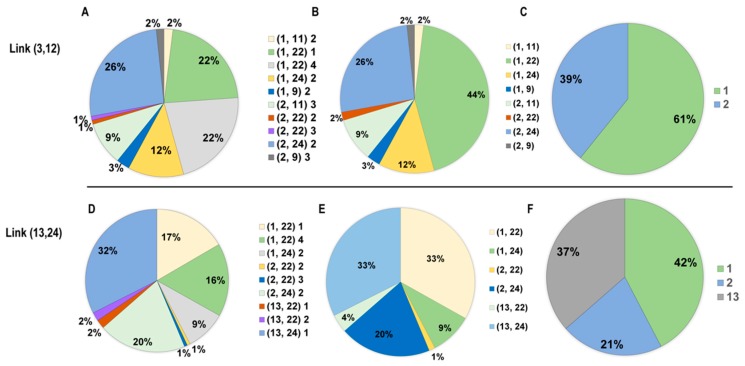
Conceptual illustration of different layers of congestion mitigation; (**A**) The path flows that contribute to the volume on link (3,12); (**B**) The OD volumes that contribute to the volume on link (3,12); (**C**) The trip generations that contribute to the volume on link (3,12); (**D**) The path flows that contribute to the volume on link (13,24); (**E**) The OD volumes that contribute to the volume on link (13,24); (**F**) The trip generations that contribute to the volume on link (13,24).

Because the CG-based learning framework internally integrates the four-step process of transportation modeling and enables the estimation of the flows on all paths in the candidate path set, traffic managers can apply the results to analyze the individual components of the congestion on each link. Figure 8 displays the congestion pie charts for links (3, 12) and (13, 24).

Table 4 further shows how the volume on link (3, 12) is composed of 10 path flows. As shown in Figure 8A and Table 4, the following three paths (bolded in Table 4) contribute the majority (a total of 70%) of the traffic volume on link (3, 12):

- 2→1→3→12→13→24.
- 1→3→12→13→24→21→22.
- 1→3→12→13→24→23→22.

Furthermore, by simply combining the corresponding path flows, it can be seen from Figure 8B and Table 4 that link (3, 12) is mainly passed by users of OD pairs (2, 24), (1, 22), and (1, 24) (26%, 44%, and 12%, respectively). Figure 8C and Table 4 show that 61% of the flow on link (3, 12) is generated from node 1. We can analyze the congestion components for link (13, 24) in a similar manner; see Table 5 and Figure 8D–F. The pie charts show that 37% of the flow on link (13, 24) is generated from node 13 and that most of the corresponding paths terminate at node 24. The detailed congestion components are presented in Table 4.

This congestion component analysis provides us with useful information about the sources of congestion. Knowledge of the congestion pie charts is very important for selecting the zones, OD pairs, or paths where congestion mitigation strategies will have the greatest effect. For example, if we wish to decrease the flows on link (3, 12), relocating some workplaces at node 22 to other zones might be an effective method.

#### 5.1.3. Marginal Effect Analysis

**Figure 9:**
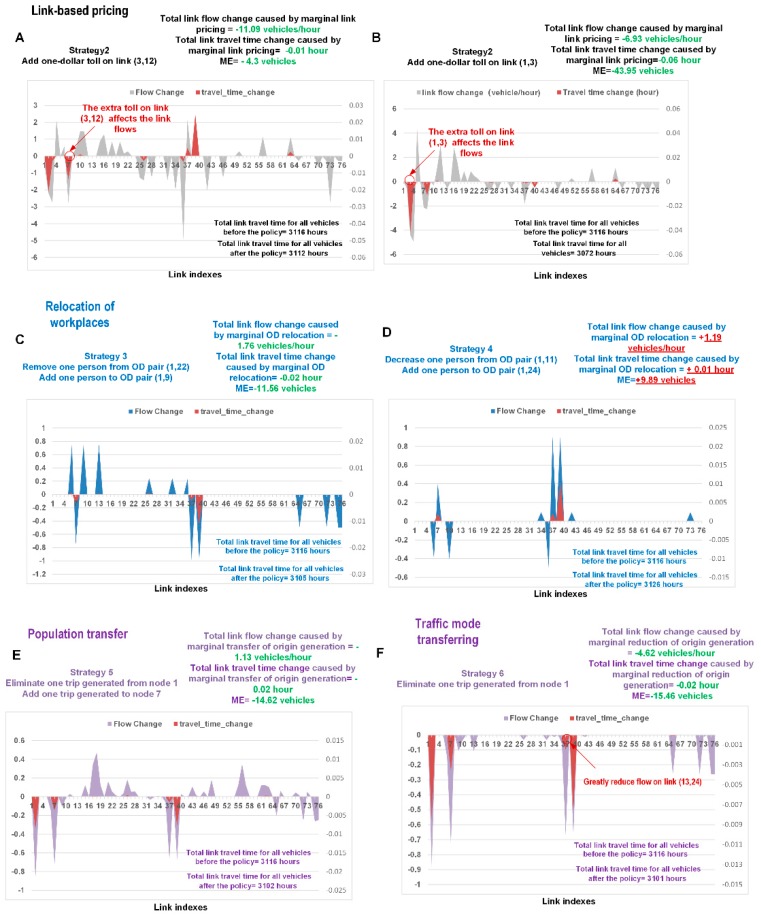
Marginal effect (ME) analyses of different congestion mitigation strategies; (**A**) ME analyses of adding one-dollar toll on link (3, 12); (**B**) ME analyses of adding one-dollar toll on link (1, 3); (**C**) ME analyses of removing one person from OD pair (1,22) and adding one person to OD pair (1,9); (**D**) ME analyses of removing one person from OD pair (1,11) and adding one person to OD pair (1,24); (**E**) ME analyses of removing one person from zone 1 and adding one person to zone 7; (**F**) ME analyses of removing one person from zone 1.

In this experiment, we aimed to evaluate the MEs of various congestion mitigation strategies. We used the Bureau of Public Roads (BPR)-form function as the travel time function, F(v), in Equation (36). The current estimated link flows and the parameters of the BPR function are reported in Appendix A. Figure 9 shows the changes in the flows and travel times on each link. The MEs of the six strategies are displayed in Figure 9.

Several interesting observations can be found:

1. In Figure 9A,B, the toll successfully decreases the flows and travel times on links (3, 12) and (1, 3). It also reduces the total travel time on all links. Interestingly, although both links (3, 12) and (1, 3) are congested (465 vehicles/hour and 583 vehicles/hour, respectively), imposing a toll of one extra dollar on link (1, 3) produces more benefit than doing the same on link (3, 12). The former policy reduces the number of vehicles in the system by 44 (ME = 44 vehicles), while the latter results in a decrease of only 4 vehicles (ME = 4 vehicles). These findings demonstrate that similar pricing policies can have different effects.
2. As shown in Figure 9C, if one user changes his/her destination from node 24 to node 9, then the traffic flows on links (3, 12), (12, 13), and (13, 24) will decrease (by 0.7 vehicles/hour, 0.99 vehicles/hour, and 0.99 vehicle/hour, respectively). This figure justifies the importance of job-housing balancing in urban planning.
3. We also find that the impacts of the policies on the flows and travel times are complex, with some mitigation strategies potentially decreasing the overall welfare of the system. For the scenario depicted in Figure 9D, the ME corresponds to an increase of 9.89 vehicles in the system. The policy actually decreases the flow levels (by approximately 0.4 vehicles/hour) on links (3, 4), (4, 5), and (12, 11) (links 6, 9, and 36, respectively, in the plot); however, these three links are not approaching their capacities in their current states (257.3 vehicles/hour, 116 vehicles/hour, and 216 vehicles/hour, respectively; see the Appendix A). Unfortunately, the strategy also guides additional traffic flows (approximately 0.9 vehicles/hour) to links (12, 13) and (13, 24), which are already congested (391 vehicles/hour and 615 vehicles/hour, respectively). This is the reason why, in the short term, sometimes the functional relocation of a metropolitan area can sometimes lead to a worse result than before.
4. Figure 9E shows that the strategy of “population transfer” achieves good performance in relieving traffic congestion. As seen in Figure 9F, if methods are implemented to make fewer people from zone 1 use private cars, this strategy will also apparently increase the overall utility of the system. In particular, these policies greatly reduce the flow on the congested link (13, 24). The reason for the beneficial effects of these policies can be identified from the congestion component pie chart shown in Figure 9: In total, 42% of the flow on link (13, 24) is generated from node 1.

### 5.2. Application Study in a Beijing Subnetwork

To better demonstrate the applicability of the proposed CG-based learning framework, a median-scale experiment was conducted based on a subnetwork of Beijing with 2502 nodes, 236 zones, 14,967 OD pairs, 40,494 paths, and 5397 links. The traffic demand outside the subnetwork was merged in the traffic zones near the boundaries. We did not use mobile phone data or floating car data in this experiment. We utilized only the reference measurements for the trip generation results and OD split rates provided by the metropolitan planning organization. Loop detector data (from one hour on 113 links) from this organization were also applied. Hence, the objective function, $\min F_{1}\left(\alpha \right)+F_{2}\left(\gamma \right)+F_{4}\left(v\right)$, was used to integrate the data sources into the learning process.

The maximum number of iterations was set to 1000, and the initial learning rate was set to 0.00001. The procedure was run under Linux on a Dell PowerEdge T630 tower server with two Intel Xeon Quad CPUs, eight 16 GB of RAM, and 512 GB of SSD storage. The number of variables in the CG can be estimated as follows: Number of neurons in each layer + number of connections between layers ((236 + 14,967 + 40,494 + 5,397) + (14,967 + 40,494 + 40,494 × 5397) = 116,555). The total CPU time required for the learning process was 2 hours.

**Figure 10:**
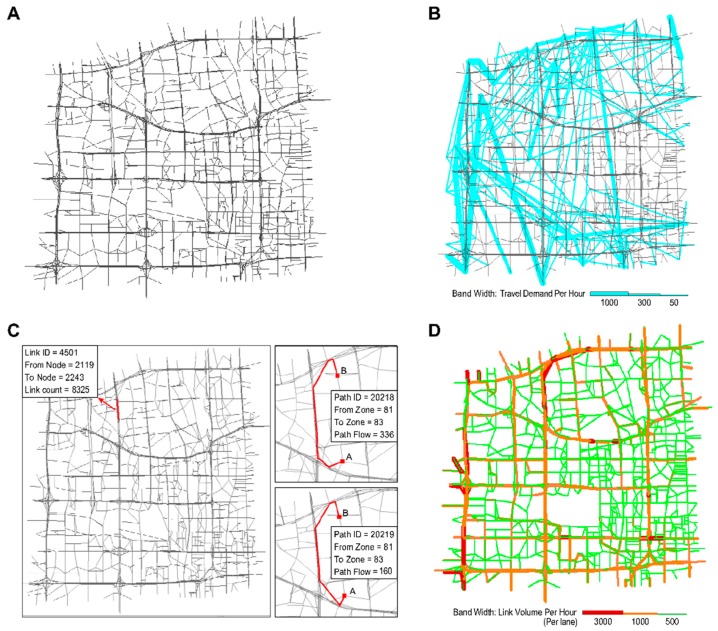
Experimental results based on the Beijing subnetwork; (**A**) physical network; (**B**) Estimated trip distribution; (**C**) The top 2 path flows contributing to the volume on link 4501; (**D**) Estimated link flows.

**Figure 11:**
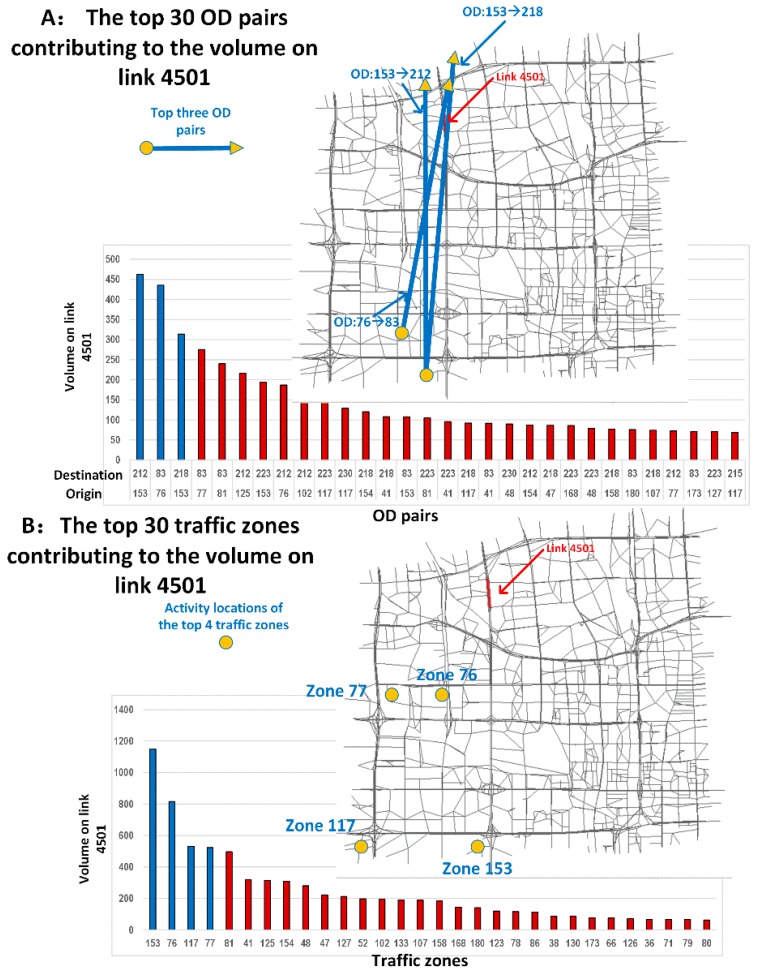
(**A**)The top 30 OD pairs in the subnetwork with the highest estimated contributions to the flow volume on link 4501; (**B**) The top 30 traffic zones in the subnetwork with the highest estimated contributions to the flow volume on link 4501.

**Figure 12:**
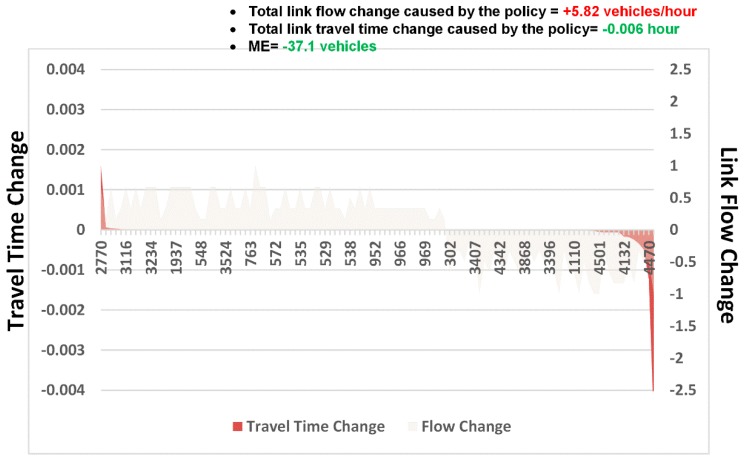
ME analysis of moving a workplace in zone 83 to zone 117.

**Figure 13:**
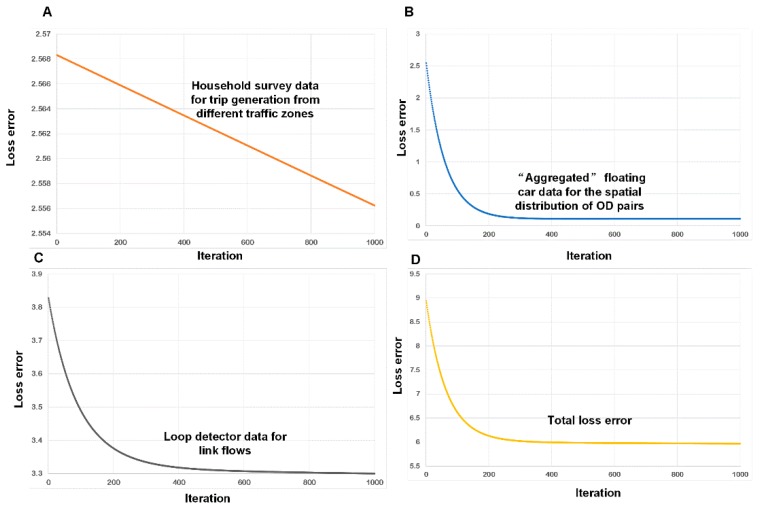
Evolution of the error values of the loss functions over 1000 iterations; (**A**) the loss error of household survey data; (**B**) the loss error of floating car data; (**C**) the loss error of link counts; (**D**) the total loss error.

Several interesting results were obtained after the experiment:

1. Estimated outputs: Figure 10A shows the physical network. The other panels in this figure illustrate the outputs estimated using different layers of the CG. Figure 10B displays the estimated trip distribution. As displayed in Figure 10D, the estimated link flows per hour per lane were also obtained using the proposed learning framework.
2. Congestion analysis: Figure 10C shows one of the most congested links (i.e., link 4501, from node 2119 to node 2243). The estimated link flow is 8325 vehicles/hour. Based on the CG, there are a total of 2157 paths passing through link 4501. Figure 10C displays the two paths that most strongly contribute to congestion on this link (paths 20218 and 20219). Furthermore, the flow on the link comes from 557 OD pairs and 102 traffic zones. Figure 11A,B display the top 30 OD pairs and trip-generating traffic zones that contribute the most to the flow volume on link 4501. We also labelled the OD pairs and traffic zones associated with the largest volume on the map. We find that the congestion on link 4501 is primarily caused by traffic demands for travel from the southern urban area to the northern subarea.
3. ME analysis: Because link 4501 and zone 83 are near several universities and companies in Beijing, one possible congestion mitigation strategy is to move some workplaces from zone 83 to zone 117. We can calculate the ME of this policy as follows. Figure 12 shows the changes in travel times and volumes on related links. Interestingly, this strategy actually increases the total volume on the links. However, it can reduce the travel times on certain highly congested links. The ME is −37.1 vehicles, which implies that the policy can indeed mitigate congestion in the traffic system.
4. Calibration: The estimation processes using different data sources are shown in Figure 13. During the experiment, we normalized the estimates and the references to lie within the range of [0, 10]. We found that the different objective functions can be simultaneously estimated during the learning process.

## 6. Conclusions and Future Research Plans

In a traffic system, congestion and inefficiency are fundamentally related to the relative balance between demand and supply. A traffic system can operate efficiently if the traffic demand can be accurately estimated. This paper proposed an integrated framework that combines traditional model-driven traffic demand estimation with emerging data-driven approaches based on a CG framework. This CG-based learning framework might contribute to filling the two research gaps mentioned in Section 1. First, the framework can help to overcome challenges related to data mining and fusion when processing big data from multiple heterogeneous sources. Second, congestion mitigation strategies can be integrated with the CG to evaluate the benefits and costs of the corresponding policies. A real-world case study was also presented to demonstrate the applicability of the framework.

However, the proposed framework also has some limitations that should be addressed in the future. First, we anticipate that the proposed CG-based framework can be developed to describe more realistic traffic demands. We can extend the learning framework based on the fundamental unit method [28], that is:

(42) $$\alpha =g\cdot t\left(p_{1},p_{2},\ldots ,p_{n}\right)$$

where g is a vector expressing the populations of different groups and the trip rate vector, $t\left(p_{1},p_{2},\ldots ,p_{n}\right)$, expresses the relationships between trip rates and various influencing factors ($\left(p_{1},p_{2},\ldots ,p_{n}\right)$). The learning framework can be used to calibrate the related parameters. For example, suppose that the trip rate of a certain population group is impacted by the price of gas, $p_{1}$. Managers can impose gas taxes to reduce the trip rate of this group of people. When trip chain data are available, we can also apply a trip chain layer in place of the OD layer shown in 4 and reconstruct the CG based on activity-based models [80,81,82,83,84]. Second, the current framework has not fully considered several important traffic flow characteristics. We simply used the BPR function to describe the basic relationship between travel times and link flows, which is not accurate according to the fundamental diagrams in traffic flow theories. Finally, the only traffic mode considered in this research was driving in private cars. We also need to extend the CG by incorporating a mode-split layer or extending the path layer based on a supernetwork to capture both automobile and public transit networks [18]. This modification would allow managers to additionally impose strategies for adjusting the choices between different traffic modes. The final version of this demand estimation engine should serve as a decision-making support platform that can simulate transportation scenarios that cannot be practically realized on the basis of the closely interconnected relationship between traffic planners and travelers. It should have the capability of predicting and visualizing the evolution of multimodal traffic systems (including private cars, buses, and metro systems) and provide useful decision support advice for managers.

## Figures and Tables

**Table 1:** Utility and probability calculations with transit as the base alternative.

Alternative	Utility		Exponent	Probability
	Expression	Value		
Drive alone	$U_{DA}=\beta_{DA,1}INC-\beta_{2}TT_{DA}$ =0.004×50−0.02×30	−0.4	0.6703	$P_{DA}=0.65$
Transit	$U_{TR}=\beta_{TR,1}INC-\beta_{2}TT_{TR}$ =0×50−0.02×50	−1	0.3679	$P_{TR}=0.35$
	$\beta_{DA,1}=0.004$; ${\;\beta }_{TR,1}=0;{\;\beta }_{2}=-0.02$		∑=1.0382	

**Table 2:** Mapping of congestion mitigation strategies in the multilayer CG framework.

Policy	Layer in the CG	Purpose	Variable	1/Δ
Population transfer/ taxation	Trip generation layer	Reduce the number of users in a zone Reduce users’ trip rates	α	$\frac{\partial v}{\partial \alpha }=\frac{\partial v}{\partial f}\frac{\partial f}{\partial q}\frac{\partial q}{\partial \alpha }$
Urban functional re-layout	Trip distribution layer	Jobs-housing balance Reduce users’ travel distances	q	$\frac{\partial v}{\partial q}=\frac{\partial v}{\partial f}\frac{\partial f}{\partial q}$
Relocation of workplaces	Trip distribution layer	Jobs-housing balance Reduce users’ travel distances	γ	$\frac{\partial v}{\partial \gamma }=\frac{\partial v}{\partial f}\frac{\partial f}{\partial q}\frac{\partial q}{\partial \gamma }$
Traveler information provision	Path flow layer	Change users’ route choice behaviors	ρ	$\frac{\partial v}{\partial \rho }=\frac{\partial v}{\partial f}\frac{\partial f}{\partial \rho }$
Link-/path-based pricing/credit	Path/link flow layer	Change users’ route choice behaviors	TC	$\frac{\partial v}{\partial \mathrm{TC}}=\frac{\partial v}{\partial f}\frac{\partial f}{\partial \rho }\frac{\partial \rho }{\partial \mathrm{TC}}$
Infrastructure improvement	Path/link flow layer	Change users’ route choice behaviors	TT	$\frac{\partial v}{\partial \mathrm{TT}}=\frac{\partial v}{\partial f}\frac{\partial f}{\partial \rho }\frac{\partial \rho }{\partial \mathrm{TT}}$

**Table 3:** Comparison of our CG-based learning framework and the Kalman Filter (KF).

Model	Computational Graph (CG)	Kalman Filter (KF)
State variables	Trip generation Trip distribution Path/link flows	Traffic state variables OD volume
Traffic observations	Multiple data sources	Time-varying observations
Algorithm process	Recursive forward/backward propagation	Recursive prediction and updating
Update method	Gradient	Kalman optimal gain
Noise	Stochastic gradient descent	Gaussian distribution
Control inputs	Policies imposed offline	External influences imposed online
Correlation between variables	Composite function	Covariance matrix
State transitions	Layer-based nonlinear transitions	Stage-based linear transitions

**Table 4:** Analysis of congestion components for link (3, 12) (units: vehicles/hour).

From Zone	To Zone	Path Index	Node Sequence	Contributed Path Flow	Contributed OD Volume	Contributed Zone Production
1	9	2	1→3→12→11→10→9	13.4	13.4	282.9
1	11	2	1→3→12→11	9.1	9.1	
1	22	1	**1→3→12→13→24→21→22**	**102**	203.7	
1	22	4	**1→3→12→13→24→23→22**	**101.8**		
1	24	2	1→3→12→13→24	56.7	56.7	
2	9	3	2→1→3→12→11→10→9	7.8	7.8	182.5
2	11	3	2→1→3→12→11	44.4	44.4	
2	22	2	2→1→3→12→13→24→23→22	4.2	8.4	
2	22	3	2→1→3→12→13→24→21→22	4.2		
2	24	2	**2→1→3→12→13→24**	**122**	122	
			Link volume	465.4		

**Table 5:** Analysis of congestion components for link (13, 24) (units: vehicles/hour).

From Zone	To Zone	Path Index	Node Sequence	Contributed Path Flow	Contributed OD Volume	Contributed Zone Production
1	22	1	1→3→12→13→24→21→22	102	203.7	260.4
1	22	4	1→3→12→13→24→23→22	101.8		
1	24	2	1→3→12→13→24	56.7	56.7	
2	22	2	2→1→3→12→13→24→23→22	4.2	8.4	130.3
2	22	3	2→1→3→12→13→24→21→22	4.2		
2	24	2	2→1→3→12→13→24	122	122	
13	22	1	13→24→21→22	12.3	24.5	224.1
13	22	2	13→24→23→22	12.3		
13	24	3	13→214	199.6	198.6	
			Link volume	612.1		

## Appendix A

The current estimated link flows and parameters of the Bureau of Public Roads (BPR) function are shown in Table A1 The BPR function used to evaluate the MEs is as follows:

(A1) $$F\left(v\right)=\frac{\mathrm{Link}\;\mathrm{length}}{\mathrm{Free}\;\mathrm{flow}\;\mathrm{speed}}\left(1+1.5{\left(\frac{\mathrm{Link}\;\mathrm{flow}}{\mathrm{Capacity}}\right)}^{4}\right)$$

**Table A1:** Current estimated link flows and parameters of the Bureau of Public Roads (BPR) function.

Link ID	Link Name	Estimated Flow (vehicles/hour)	Length (mile)	Free Flow Speed (miles/hour)	Capacity (vehicles/hour)
1	(1, 2)	0.94	6	60	300
**2**	(1, 3)	582.59	4	60	300
3	(2, 1)	242.51	6	60	300
4	(2, 6)	96.49	5	60	300
5	(3, 1)	0	4	60	300
**6**	(3, 4)	257.3	4	60	300
7	(3, 12)	465.36	4	60	300
8	(4, 3)	0	4	60	300
**9**	(4, 5)	115.66	2	60	300
10	(4, 11)	213.08	6	60	300
11	(5, 4)	71.45	2	60	300
12	(5, 6)	0	4	60	300
13	(5, 9)	177.53	5	60	300
14	(6, 2)	0	5	60	300
15	(6, 5)	119.96	4	60	300
16	(6, 8)	42.75	2	60	300
17	(7, 8)	244.9	3	60	300
18	(7, 18)	245.92	2	60	300
19	(8, 6)	66.22	2	60	300
20	(8, 7)	17	3	60	300
21	(8, 9)	158.5	10	60	300
22	(8, 16)	45.93	5	60	300
23	(9, 5)	13.36	5	60	300
24	(9, 8)	0	10	60	300
25	(9, 10)	27.45	3	60	300
26	(10, 9)	376.53	3	60	300
27	(10, 11)	45.45	5	60	300
28	(10, 15)	30.2	6	60	300
29	(10, 16)	0	5	60	300
30	(10, 17)	0	8	60	300
31	(11, 4)	0	6	60	300
32	(11, 10)	153.29	5	60	300
33	(11, 12)	0	6	60	300
34	(11, 14)	17.99	4	60	300
35	(12, 3)	140.07	4	60	300
**36**	(12, 11)	215.78	6	60	300
37	(12, 13)	390.75	3	60	300
38	(13, 12)	281.24	3	60	300
39	(13, 24)	614.86	4	60	300
40	(14, 11)	78.82	4	60	300
41	(14, 15)	0	5	60	300
42	(14, 23)	17.99	4	60	300
43	(15, 10)	117.69	6	60	300
44	(15, 14)	6.83	5	60	300
45	(15, 19)	0	4	60	300
46	(15, 22)	30.2	4	60	300
47	(16, 8)	0	5	60	300
48	(16, 10)	153.75	5	60	300
49	(16, 17)	0	2	60	300
50	(16, 18)	17.86	3	60	300
51	(17, 10)	0	8	60	300
52	(17, 16)	0	2	60	300
53	(17, 19)	0	2	60	300
54	(18, 7)	78.78	2	60	300
55	(18, 16)	125.67	3	60	300
56	(18, 20)	204.05	4	60	300
57	(19, 15)	58.78	4	60	300
58	(19, 17)	0	2	60	300
59	(19, 20)	0	4	60	300
60	(20, 18)	144.72	4	60	300
61	(20, 19)	58.78	4	60	300
62	(20, 21)	306.22	6	60	300
63	(20, 22)	333.84	5	60	300
64	(21, 20)	0	6	60	300
65	(21, 22)	292.64	2	60	300
66	(21, 24)	132.01	3	60	300
67	(22, 15)	65.74	4	60	300
68	(22, 20)	0	5	60	300
69	(22, 21)	0	2	60	300
70	(22, 23)	59.1	4	60	300
71	(23, 14)	71.99	4	60	300
72	(23, 22)	118.18	4	60	300
73	(23, 24)	77.09	2	60	300
74	(24, 13)	0	4	60	300
75	(24, 21)	118.42	3	60	300
76	(24, 23)	190.17	2	60	300
